# Biliary and Vascular Complications after Liver Transplantation–From Diagnosis to Treatment

**DOI:** 10.3390/medicina59050850

**Published:** 2023-04-28

**Authors:** Gina Gheorghe, Camelia Cristina Diaconu, Simona Bungau, Nicolae Bacalbasa, Natalia Motas, Vlad-Alexandru Ionescu

**Affiliations:** 1 Faculty of Medicine, University of Medicine and Pharmacy Carol Davila Bucharest, 050474 Bucharest, Romania; gina.gheorghe@drd.umfcd.ro (G.G.); nicolae.bacalbasa@umfcd.ro (N.B.); vladalexandru.ionescu92@gmail.com (V.-A.I.); 2 Gastroenterology Department, Clinical Emergency Hospital of Bucharest, 105402 Bucharest, Romania; 3 Internal Medicine Department, Clinical Emergency Hospital of Bucharest, 105402 Bucharest, Romania; 4 Department of Visceral Surgery, Center of Excellence in Translational Medicine “Fundeni” Clinical Institute, 022328 Bucharest, Romania; 5 Department of Pharmacy, Faculty of Medicine and Pharmacy, University of Oradea, 410028 Oradea, Romania; 6 Institute of Oncology “Profesor Doctor Alexandru Trestioreanu” Bucharest, 022328 Bucharest, Romania; natalia.motas@umfcd.ro; 7 Department of Thoracic Surgery, University of Medicine and Pharmacy Carol Davila Bucharest, 050474 Bucharest, Romania

**Keywords:** liver transplantation, biliary complications, vascular complications, graft dysfunction

## Abstract

The last decades have brought impressive advances in liver transplantation. As a result, there was a notable rise in the number of liver transplants globally. Advances in surgical techniques, immunosuppressive therapies and radiologically guided treatments have led to an improvement in the prognosis of these patients. However, the risk of complications remains significant, and the management of liver transplant patients requires multidisciplinary teams. The most frequent and severe complications are biliary and vascular complications. Compared to vascular complications, biliary complications have higher incidence rates but a better prognosis. The early diagnosis and selection of the optimal treatment are crucial to avoid the loss of the graft and even the death of the patient. The development of minimally invasive techniques prevents surgical reinterventions with their associated risks. Liver retransplantation remains the last therapeutic solution for graft dysfunction, one of the main problems, in this case, being the low number of donors.

## 1. Introduction

Patients with primary liver tumors or acute liver failure may benefit from liver transplantation as a therapeutic option, in addition to those with end-stage liver disease [1,2]. Since 1963, when the first human liver transplant was performed, significant advancements have been made in the treatment of patients who are candidates for liver transplantation, leading to a better prognosis [1,2,3,4]. Currently, the survival rates of recipients of a transplanted liver vary between 91.8% at 1 year and 75.1% at 5 years [5].

Despite the remarkable progress registered in preoperative assessment, surgical technique, postoperative care, immunosuppression or antiviral therapy, the postoperative morbidity and mortality rates remain high [1]. The main complications are biliary complications, that affect up to a quarter of recipients [1]. There are also differences regarding the incidence of biliary complications depending on the type of donor (donation after circulatory death -DCD, donation after brain death -DBD and living donation -LD). Meier et al. reported better graft survival rates in the case of DBD and LD liver transplantation (LT) compared to DCD-LT (92.6–79.9% for DBD-LTs, 91.7–82.9% for LD-LTs, 88.6–70% for DCD-LTs) [6]. Thus, patients with DCD-LTs had a 1.7–1.3 times higher risk of losing their graft compared to patients with DBD-LTs and LD-LTs [6]. However, the highest rates of bile leaks were recorded in patients with LD-LTs (36.2% LD-LTs, 10.1% DCD-LTs and 7.2% DBD-LTs) [6]. Non-anastomotic biliary stenosis was present in 15.2% of patients with DCD-LTs, 4.3% of those with LD-LTs, and 1.4% of those with DBD-LTs [6]. The use of machine perfusion techniques can lead to the reduction in potential complications that may appear in patients with DCD-LT [7]. Machine perfusion is a promising method that allows the extension of the donor base (DCD and extended criteria donor (ECD)–from patients over 60 years old or patients between 50 and 59 years old with vascular comorbidities) [7]. Hypothermic, subnormothermic, normothermic machine perfusion techniques have been shown to significantly improve liver damage in DCD livers compared to static cold storage (SCS) in animal models [7]. Currently, there is no consensus regarding the use of machine perfusion for ECD livers, but recent data supports the reduction in ischemic reperfusion injury to the liver graft [7]. The use of machine perfusion reduces the level of proinflammatory cytokines and the expression of genes that control the phenomenon of hypoxia during reperfusion [7]. Additionally, machine perfusion prevents Kupffer and epithelial cell activation and reduces recipient T-cell infiltration of the graft [7]. Olschewski et al. reported an improvement in liver viability after prolonged warm ischemic damage by using machine perfusion [8]. The same authors highlighted that the perfusion temperature of 21 °C leads to a significant reduction in portal venous resistance and an increase in bile production [8]. Another study demonstrated that subnormothermic machine perfusion allows the restoration of liver graft viability by restoring mitochondrial function and improving ion and metabolite balances [9]. Additionally, subnormothermic machine perfusion allows the reduction of microcirculatory obstructions secondary to microthrombi or cellular edema [9].

Vascular complications occur less frequently but are associated with high morbidity and mortality rates [10,11]. Currently, vascular complications represent the second most common cause of liver graft failure, after graft rejection, being associated with increased death rates [12,13]. Approximately 7% of patients with liver transplants from deceased donors and 13% of those with liver transplants from living donors have been documented to have vascular complications [2,13,14,15].

The early detection and treatment of potential complications are critical for graft maintenance and improving the prognosis of liver transplant recipients [16,17,18].

## 2. Biliary Complications

Biliary complications are a significant cause of morbidity and mortality after liver transplantation [19,20]. In the case of transplantation from a deceased donor, the incidence of biliary complications ranges from 10% to 15%, whereas it ranges from 15% to 30% in the case of transplantation from a living donor [21,22].

Most biliary complications, classified as biliary strictures or biliary fistulas, develop within the first six months following transplantation. Since the biliary tree is sensitive to the lowering of hepatic artery flow, the identification of a biliary complication requires a careful assessment of the hepatic artery [2,23]. Depending on the moment of their appearance, biliary complications are divided into early complications (occurring within 30 days after liver transplantation) and late complications (occurring more than 30 days after liver transplantation) [2,23,24] (Table 1).

**Figure 1:**
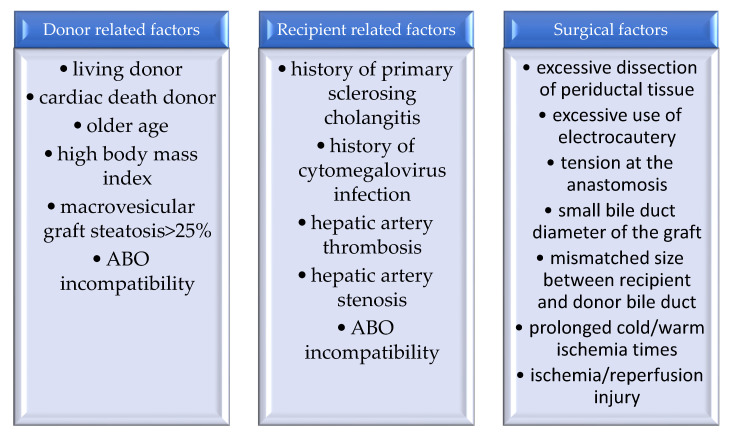
Risk factors for biliary complications after liver transplantation.

The risk factors for developing biliary complications following liver transplantation may be related to the donor, to the recipient and to the surgical procedure (Figure 1) [25,26].

### 2.1. Biliary Strictures

Around 40% of all biliary complications are biliary strictures, the most common complications after liver transplantation [27,28]. In the case of transplantation from a brain-dead donor, the incidence of biliary strictures ranges from 5 to 15%, while it ranges from 28 to 32% in the case of transplantation from a living donor [2,29]. Jarlot-Gas et al. followed a group of 225 liver transplant recipients and identified the presence of biliary strictures in 53 patients (24.9%) [2,30]. Related to the time of identification of these complications, 69.6% were diagnosed in the first 6 months after liver transplant [30].

The most significant causes of biliary strictures include the type of liver transplant procedure (living donor liver transplant > deceased donor liver transplant), surgical techniques, use of a T-tube, development of hepatic artery thrombosis and ischemic lesions of the biliary ducts [27,31]. Diffuse and multifocal strictures are often caused by the latter.

Anastomotic and non-anastomotic strictures are the two main categories of biliary strictures following liver transplantation [2,27]. A significant factor in determining the risk of biliary complications following liver transplantation is the type of biliary anastomosis [32]. The two most common biliary repair techniques are choledocho-choledochian anastomosis and choledocho-jejunal anastomosis [33]. Several factors influence the selection of the type of biliary reconstruction, including the underlying liver pathology, dimensions of the donor and recipient bile ducts, history of previous biliary surgery and the surgical team preferences. There are currently no definite recommendations regarding the optimal type of biliary reconstruction [34]. The choledocho-choledochian anastomosis is generally preferred because of the lower technical difficulty, preservation of the sphincter of Oddi and easier subsequent endoscopic access to the biliary system [35,36]. Preservation of the sphincter of Oddi also decreases the risk of developing ascending cholangitis [35,36]. The placement of a Kehr tube at the level of the choledoco-choledochian anastomosis enables the measurement of bile flow in the early postoperative period but also maintains an easy access path for the radiological evaluation of the biliary system [36,37,38]. However, Kehr tubes have been linked with the risk of biliary fistulas and potential episodes of cholangitis at the time of their removal [36,37,38]. The rate of overall complications was higher among patients in whom this tube was fitted (33% in patients with a Kehr tube vs. 15% in patients without a Kehr tube), and the survival rate was slightly lower [35]. A meta-analysis that followed 267 studies published between 1995 and 2020 reported an increase in the incidence of overall biliary complications (*p* = 0.049), bile leaks (*p* = 0.048) and cholangitis (*p* = 0.02) among patients in whom T-tubes were used [39]. Thus, choledocho-choledochian anastomosis without Kehr tube insertion is the most frequently used surgical technique in liver transplantation. This may be associated with the subsequent development of biliary strictures in approximately 20% of cases [36,37,38].

The choledocho-jejunal anastomosis may be preferred in patients with specific pre-existing biliary diseases, such as primitive sclerosing cholangitis, those with previous biliary surgery, and also in the case of size incompatibility between the donor and recipient bile ducts [36,40]. Compared to the choledocho-choledochian anastomosis, the choledocho-jejunal anastomosis is more difficult to approach later by endoscopy [40]. Among the potential complications of choledocho-jejunal anastomosis are intestinal perforation, development of strictures, fistulas or hemorrhages at the level of anastomosis [40,41].

Clinically, patients who develop biliary strictures may present with jaundice, fever and abdominal pain [42]. The paraclinical biochemical evaluation can highlight cholestasis syndrome, and imaging evaluation may reveal dilation of the bile ducts (without being a mandatory condition for establishing a positive diagnosis) [42]. There are also patients with bile duct strictures, without clinical symptoms, but with cholestasis syndrome at the paraclinical evaluation [42]. The histopathological examination can suggest a biliary obstruction by identifying pericholangitis or cellular proliferation in the bile ducts [43].

Anastomotic biliary strictures must be differentiated from non-anastomotic biliary strictures, which are mainly due to ischemic lesions of the biliary epithelium and immunological reactions [44,45]. Non-anastomotic biliary strictures are defined as strictures involving the donor’s common hepatic duct proximal to the anastomosis, right and/or left hepatic duct, and/or intrahepatic branches [36,44]. These are usually multiple, complex and longer, representing approximately 5–10% of all biliary complications [45].

The majority of strictures are nowadays treated with endoscopic retrograde cholangiopancreatography (ERCP), which involves dilation and stenting at the level of the stricture, as opposed to the initial years following the introduction of liver transplantation, when roughly 70% of patients who experienced biliary complications returned to the operating room for therapeutic management [2,46,47,48]. Repeated sessions of dilations and biliary stenting are usually required to achieve an effective therapeutic response. Calibration of the stenotic area is aimed at each ERCP treatment session. This objective is achieved by increasing the number or diameter of the biliary prostheses used (plastic stents) successively at each session. This therapeutic plan lasts approximately one year [46,47,48,49]. The number of endoscopic dilation sessions also depends on the type of stenosis. Thus, in the case of early anastomotic strictures, defined as strictures that appear in the first two months after liver transplantation, a single session of endoscopic balloon dilatation or/and plastic stent placement is usually required [50]. In most of these cases, the stricture resolves within three months, with no further interventions required to maintain a patent anastomosis [50]. In the case of late anastomotic strictures, defined as strictures that appear no later than two months after transplantation, repeated endoscopic dilation sessions are usually necessary [51]. The interval at which biliary stents are changed is three months, to avoid stent occlusion and bacterial cholangitis [2,51,52]. More than 80% of cases of biliary strictures have been successfully managed with endoscopic procedures [49].

In patients with choledocho-jejunal anastomosis, a balloon enteroscope or, alternatively, percutaneous transhepatic drainage and subsequent biliary stent insertion may be required to reach the anastomosis [2,53,54].

In the case of non-anastomotic strictures, the endoscopic treatment is more complex, as they are usually multiple and diffuse. The long-term efficiency of endoscopic therapy, based on balloon dilation and/or the placement of plastic biliary strictures, is approximately 50% [55,56]. Some of these patients may require percutaneous transhepatic cholangiography or surgical reintervention [57]. Additionally, in these cases, there is a greater risk of losing the liver graft and the need for retransplantation [58].

The use of self-expanding metal stent (SEMS) appears to be increasing for ERCP treatment of anastomotic biliary strictures [2]. In the case of strictures secondary to ischemic lesions, their frequently proximal location to the anastomosis greatly limits the use of SEMS. Recent data suggest that fully covered self-expandable metallic stents (cSEMS) could significantly reduce the number of endoscopic interventions, with an efficacy similar to the sequential placement of plastic stents [2]. However, the optimal duration of cSEMS therapy is not clearly established, and the recurrence of stenosis appears to be higher in the cSEMS patient groups than in the sequential plastic stent patient groups [2,59,60].

A closer look at the studies that evaluated the rate of stenosis recurrence led to conflicting results. Data from the literature have thus reported similar recurrence rates for cSEMS and plastic stents [61,62]. Regarding all the possible complications and rate of stent migration, no significant differences were identified between SEMS and plastic stents [61,62].

In the absence of a response to endoscopic or percutaneous treatment, surgical intervention may be indicated. Previous endoscopic or percutaneous treatment has not been shown to influence the success rate of surgery for these complications [33].

After the resolution of the stricture, a periodic clinical and biological evaluation of these patients is necessary. Thus, every three months serum levels of alanine amino-transferase, aspartate aminotransferase, alkaline phosphatase and total bilirubin are measured [63]. If elevated values of these biomarkers are identified, paraclinical investigations are continued by performing magnetic resonance cholangiography [63].

### 2.2. Biliary Fistulas

Biliary fistulas are the second most common biliary complication of liver transplantation, after biliary strictures [10]. According to the literature data, the incidence rate of these complications varies between 2 and 25% [64,65]. Among the risk factors that increase the risk of biliary fistulas are those related to surgical technique (tension of the anastomosis, excessive use of diathermy, incomplete cystic stump suture, cut surface of the graft, premature T-tube extraction) and ischemic injury [2,66,67,68]. The factors unrelated to Kehr tube removal typically occur within the first 30 days following transplantation [32,66,67,68]. In total or partial graft recipients from a brain-dead donor, most biliary fistulas are located in the anastomoses and may present as biliomas or diffuse biliary peritonitis [66,67,68]. In recipients of total or partial grafts from living donors, biliary fistulas can occur along the margin of the liver section [32,66,67,68]. The occurrence of a biliary fistula represents an independent risk factor for the emergence of biliary strictures [33].

Biliary fistulas are also classified into two large categories: anastomotic and non-anastomotic [10]. The most common ones are anastomotic fistulas, which usually develop in the first four weeks after liver transplantation [33,69].

Clinically, patients who have developed biliary fistulas may present abdominal pain, fever or any clinical sign suggestive of peritonitis. Paraclinically, the biochemical tests can reveal increased values of serum bilirubin, and imaging examinations, externalization of bile, ascites or fluid collections. ERCP with cholangiographic examination may show contrast fluid extravasation at the site of the biliary fistula [70,71]. Some patients, especially those receiving corticosteroid treatment, may be asymptomatic. In this case, the unexplained increase in the serum level of bilirubin, fluctuation of the serum level of immunosuppressive drugs or the presence of biliary ascites should immediately raise the suspicion of biliary fistula. The fistulous orifice is typically found at the biliary anastomosis level and may be caused by ischemic lesions or incomplete healing of the anastomotic segment [70].

The treatment of a biliary fistula is based on ERCP with sphincterotomy, correction of the defect by biliary stenting, which enables the biliary tree to decompress and the fistulizing region to spontaneously close, or nasobiliary drainage. These techniques can be used independently or in combination [2,71]. The resolution of biliary fistulas may be associated with secondary development of biliary strictures, because of chronic inflammatory response during the healing period [1]. In these conditions, it is recommended to maintain biliary prostheses for several months, to prevent stenotic complications [1]. Treatment-refractory biliary fistulas are associated with reduced survival [1].

Sludge and gallstones occur in approximately 5–10% of liver transplant patients and are typically linked to other biliary complications, most frequently biliary strictures [32,71,72,73]. Sludge and gallstones develop because of a higher bile viscosity and decreased bile flow [73]. Additionally, drugs such as cyclosporine may contribute to lithogenesis because of a reduced bile secretion, with biliary stasis [73,74,75,76,77]. In patients undergoing liver transplants, bile is supersaturated with cholesterol [74,75]. These patients may present cholangitis-related signs and symptoms, such as abdominal pain, with cholestasis syndrome [73,74].

The presence of simple gallstones benefits from ERCP with their extraction [76]. In patients with gallstones developed secondary to biliary strictures, it is necessary, prior to the endoscopic extraction of the stones, to treat the stenotic area by dilation or stenting. In addition, patients undergoing progressive replacement of biliary stents are prone to the development of biliary sludge. In these conditions, reducing the interval of changing biliary prostheses may be necessary for the consequent reduction in the risk of developing biliary lithiasis/sludge [33]. Digital cholangioscopy allows the controlled fragmentation of large gallstones, reducing the risk of damage to the biliary epithelium [77]. In complex cases, advanced techniques, such as electrohydraulic lithotripsy or Holmium laser, can be used [77,78].

### 2.3. Bilioma and Abscess

Biliomas can form because of bile extravasation into the liver or abdominal cavity. Their secondary infection will lead to abscesses. A small bilioma communicating with the biliary tree can resorb spontaneously [79]. In contrast, large biliomas and abscesses require antibiotic treatment and percutaneous drainage. In some cases, stenting of the extrahepatic bile ducts may be necessary [79]. The last therapeutic resource remains the surgical drainage of the bilioma/abscess [79].

Sphincter of Oddi dysfunction (SOD) can occur in 1–7% of liver transplant patients [67]. Among the etiological factors are chronic injury, fibrotic tissue formation and denervation of the common bile duct [4,80]. According to Milwaukee classification, there are three types of SOD:

- Type I, true sphincter stenosis.
- Type II, the association of a structural dysfunction with motility disorder.
- Type III, functional biliary type pain [15,81]. Roma III criteria sustain the elimination of SOD type III and replacement with the terminology “functional biliary or pancreatic SOD” [68]. Biliary manometry can be used for diagnosis. The treatment of this condition usually involves only endoscopic sphincterotomy and in case of fibrosis of the sphincter apparatus, placement of stents [80,81,82,83].

## 3. Vascular Complications

Vascular complications after liver transplantation are uncommon, but they are among the most serious, with high rates of graft loss and even death (Table 2) [10,20]. An early diagnosis and appropriate therapeutic management of vascular complications are critical in these conditions, for better transplant success rates [10,13].

The most used imaging method for vascular assessment remains Doppler ultrasonography (US) [10]. This investigation can be performed immediately after liver transplantation, when other imaging techniques are not adequate [13]. Contrast ultrasound also has an important diagnostic role, allowing a better visualization of the vessels and differentiation between occlusion and stenosis of the hepatic artery [84]. Contrast-enhanced computed tomography (CT) can be used when the ultrasound evaluation results are ambiguous or do not correspond to the clinical suspicion, but it can also be used when a more detailed anatomical vascular evaluation is required to plan an endovascular intervention [9]. Catheter angiography can be used when a hepatic artery abnormality is suspected, but not confirmed by US or CT examination [13]. The role of magnetic resonance imaging (MRI) in the diagnosis of vascular complications after liver transplantation is limited to patients with renal failure [13]

All vascular complications require prompt and aggressive treatment, especially HAT and PVT [6]. These can lead to a sudden interruption of hepatic vascularization, with high rates of graft loss and retransplantation [14,85]. Currently, the therapeutic options are surgical revascularization, percutaneous angioplasty, percutaneous thrombolysis, retransplantation or conservative approach [10]. The remarkable progress made in the field of interventional radiology has resulted in radical changes in the diagnosis and treatment of vascular complications in liver transplant patients [10]. Thus, endovascular therapies such as balloon angioplasty, stenting and catheter-based thrombolytic intervention are currently the therapy of choice in most cases, replacing surgery [6,86,87].

### 3.1. Hepatic Artery Thrombosis

HAT is the most common and severe arterial complication of liver transplantation, accounting for roughly half of all arterial complications [10]. Currently, the incidence of HAT ranges between 0 and 12% and is the major cause of primary liver graft non-function [10]. In the absence of revascularization, the retransplantation rate ranges from 25 to 83%, with a death rate of up to 50% [10,88,89,90]. The clinical presentation of these patients varies from the absence of symptoms, especially if it occurs late post-transplantation, to fulminant liver necrosis [10]. The clinical expression depends on the presence of vascular collaterals that can develop from the first two weeks after transplantation [10,91,92]. Biologically, increases in the serum values of aminotransferases and bilirubin can be identified [93]. Delays in diagnosis can result in bile duct ischemia, liver necrosis, and failure, resulting in graft loss. Because the biliary tree’s vascular source is derived from hepatic artery branches, HAT can compromise biliary anastomoses, resulting in biliary strictures, chronic cholangitis, biliary abscesses, sepsis, and death [2,94]. Ultrasonographic surveillance can detect early thrombosis and lead to a significant reduction in the occurrence of biliary complications and graft loss [2,10].

The current therapeutic possibilities are endovascular or surgical revascularization, retransplantation or monitoring [2,10]. The choice of treatment is heavily influenced by the time of diagnosis [10]. Retransplantation was considered the preferred treatment, with a significant increase in survival [2,10,12]. Percutaneous endovascular treatments, such as percutaneous transluminal angioplasty and stent placement, or intraarterial thrombolysis, have shown satisfactory success over the years [2,10,87]. However, these therapeutic options remain controversial because of the risk of complications, mainly hemorrhages [10,87]. In rare cases, the development of collateral circulation allows survival even in the absence of revascularization or retransplantation [95]. This phenomenon of neovascularization was associated with four factors: early HAT, late HAT, Roux-en-Y anastomosis and site of thrombosis [92,95].

### 3.2. Portal Vein Thrombosis

Venous complications are much rarer than arterial ones, with an incidence rate that does not exceed 3% [15,96]. Acute thrombosis can cause graft dysfunction and a clinical picture that resembles fulminant liver failure [2,10]. Slower-onset thrombosis can result in portal hypertension complications such as variceal bleeding or the development of ascites [10]. The risk factors of post-transplant PVT are pre-transplant venous thrombosis, hypercoagulable states, perioperative hypotension, large porto-systemic collaterals that cause decreased portal venous flow and stasis, vessel size mismatch and graft fibrosis that disrupts the portal flow [2,10].

The most used imaging investigation that allows a noninvasive evaluation of vascular flow permeability is Doppler ultrasonography [97]. Diagnostic protocols vary between transplant centers, with some authors recommending Doppler ultrasound assessment daily or even twice a day in the immediate post-transplant period [97]. Some authors recommend using contrast-enhanced ultrasound to avoid diagnostic errors [98,99]. This investigation allows the identification of small trunks in peripheral portal branches [98,99].

Treatment depends largely on the time of presentation. The therapeutic options are represented by systemic anticoagulation, thrombolysis, endovascular therapies (transhepatic portal vein angioplasty with/without stent placement, transjugular intrahepatic portosystemic shunt (TIPS), percutaneous thrombolytic therapy), surgical reintervention and retransplantation [10]. The management of chronic PVT is focused on the complications of portal hypertension. In the event of posttransplantation PVT, both the graft and the patient have a lower survival rate [10,13].

To summarize, PVT is a rare but serious complication of liver transplantation. An early diagnosis and appropriate therapeutic management are crucial for improving the prognosis of these patients. Surgical thrombectomy is usually used in the early postoperative period. Currently, minimally invasive percutaneous treatments are considered effective treatments with a good safety profile.

## 4. Conclusions

Liver transplantation is the only curative treatment modality for a number of chronic end-stage liver diseases, as well as for some patients with acute liver failure. In recent years, this technique had an impressive development, with a significant increase in the number of liver transplantations performed worldwide [3]. Furthermore, advances in surgical techniques and immunosuppressive therapies have significantly improved the prognosis of these patients. However, liver transplantation remains a therapeutic intervention associated with a high risk of complications [2]. Two of the most common and severe complications of liver transplantation are biliary and vascular complications. If, in the case of biliary complications, the incidence rates are higher, but the prognosis can be favorable, in the case of vascular complications, the incidence rates are lower, but the severity is higher. For the early detection of potential complications and to reduce the risk of graft loss and even death of the patient, careful clinical and paraclinical monitoring is required. Currently, mini-invasive treatments prevent surgical reintervention and its risks in numerous cases. Liver retransplantation is the last therapeutic resource in case of a non-functional graft. The management of these patients by multidisciplinary teams is essential for the success of liver transplantation.

## Figures and Tables

**Table 1:** Biliary complications after liver transplantation.

Biliary Complications	Type
Biliary strictures	Anastomotic strictures Non-anastomotic strictures
Bile leaks	Anastomotic leaks Non-anastomotic leaks
Bile duct filling defects	Bile duct stones Bile duct casts
Other complications	Sphincter of Oddi dysfunction Biloma

**Table 2:** Vascular complications following liver transplantation.

Vascular Complications	Types
Arterial complications	Hepatic artery thrombosis (HAT) Hepatic artery stenosis (HAS) Hepatic artery pseudoaneurysm (HAP) Hepatic artery rupture (HAR)
Portal vein complications	Portal vein thrombosis (PVT) Portal vein stenosis (PVS)
Caval anastomosis complications	Caval resection and end-to-end cavo-caval anastomosis Piggy-back
